# Solitary Fibrous Tumour of the Mesentery: An Uncommon Site for a Rare Tumour

**DOI:** 10.7759/cureus.69011

**Published:** 2024-09-09

**Authors:** Amrapali Samadder, Pranjal Kalita, Evarisalin Marbaniang, Himraj Phukan, Caleb Harris, Jaya Mishra

**Affiliations:** 1 Pathology and Laboratory Medicine, North Eastern Indira Gandhi Regional Institute of Health and Medical Sciences (NEIGRIHMS), Shillong, IND; 2 Pathology, North Eastern Indira Gandhi Regional Institute of Health and Medical Sciences (NEIGRIHMS), Shillong, IND; 3 Radiology, North Eastern Indira Gandhi Regional Institute of Health and Medical Sciences (NEIGRIHMS), Shillong, IND; 4 Surgical Oncology, North Eastern Indira Gandhi Regional Institute of Health and Medical Sciences (NEIGRIHMS), Shillong, IND

**Keywords:** aggressive behavior, extrapleural, hemangiopericytoma, mesentery, solitary fibrous tumour, stat6

## Abstract

Solitary fibrous tumour (SFT), a unique spindle-cell neoplasm, was first identified in the pleura and was earlier named as haemangiopericytoma. The origin of SFT is not well established; however, it has recently been described as a mesenchymal neoplasm, probably arising from the ubiquitous dendritic interstitial cells. SFTs are predominantly benign tumours with a low risk of malignant transformation and are commonly seen in the intrathoracic region, seldom involving extrapleural locations. Although SFTs are extensively documented in medical literature, those originating in the mesentery are extremely uncommon. Here, we report an extremely rare case of a 31-year-old woman who presented with abdominal pain and was later diagnosed with SFT of the mesentery of the sigmoid colon based on histomorphology and immunohistochemistry studies. This case highlights that, although uncommon, such differential diagnoses need to be considered in cases of abdominopelvic swellings and must be differentiated from their histological mimickers for better patient care.

## Introduction

Solitary fibrous tumour (SFT) is an uncommon mesenchymal-derived tumour. Klemperer and Rabin [1] first observed it in the pleura, with an approximate incidence of 2.8 per 100,000 individuals [2]. However, more and more reports now state that it also originates from extrapleural anatomical areas [3]. Men and women are equally affected by SFTs, which are most frequent in adults and peak in occurrence between the ages of 40 and 70 years. SFTs typically manifest as painless, slowly expanding masses. When present in the abdominopelvic region, these tumours may cause symptoms such as urinary retention, distension, constipation, or early satiety [4].

Currently, these uncommon spindle neoplasms derived from the mesenchyme are categorized as either "malignant" or "typical" [3]. Different clinical or histological characteristics that influence the possibility of local recurrence or metastasis in SFTs of the thoracic and extrathoracic sites were reported by Sbaraglia et al. [4]. The preferred course of treatment is surgical excision, which has a near-100% five-year survival rate if the tumour is fully removed. Cranshaw et al. claimed that the sole factor affecting survival was the existence of malignant histology, with benign cases being non-fatal and malignant ones having a median survival of 59 months [5].

We report a case of an intra-abdominal SFT with high-risk aggressive behaviour arising from the serosal surface of the sigmoid colon.

## Case presentation

**Figure 1:**
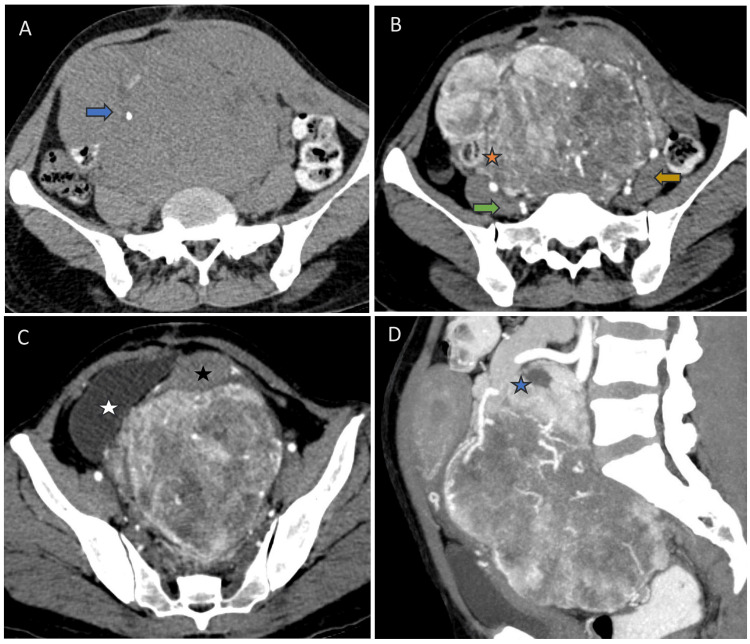
CT images. (A) Unenhanced axial scan reveals a multilobulated abdominopelvic solid lesion centred in the sigmoid mesentery with few coarse calcifications (blue arrow). (B) Contrast-enhanced axial scan in the arterial phase shows the lesion abutting the iliac arteries posteriorly with maintained fat planes (green and orange arrows). The sigmoid colon is compressed and displaced by the lesion towards the right side with no signs of obstruction (orange star). (C) The lower axial sections in arterial phase reveal the lesion abutting and displacing the uterus anteriorly (black star) and the urinary bladder to the right side (white star) with maintained fat planes. (D) Sagittal view in arterial phase shows the lesion supplied by the inferior mesenteric artery (blue star).

A 31-year-old woman presented to the Surgical Oncology OPD with complaints of a progressively increasing pelvic mass of one-year duration with on and off lower abdominal pain for the past two weeks. On physical examination, the pelvic mass was mobile, firm, and non-tender. A contrast CT abdomen revealed a heterogeneously enhanced abdominopelvic multilobulated soft-tissue lesion measuring 17.3x14.7x11.3 cm. The lesion showed multiple internal necrotic areas of non-enhancement. Anteriorly, the lesion abutted the anterior parietal peritoneum with displacement and compression of the uterus and urinary bladder. Posteriorly, it compressed the bilateral common iliac vessels and the rectosigmoid junction. There was displacement of bowel loops peripherally with sigmoid bowel loop closely enveloping the lesion on the superior and right lateral aspects with associated luminal narrowing. A clinicoradiological diagnosis of gastrointestinal stromal tumour (GIST) was favoured (Figure 1).

A decision was taken to perform an en bloc resection with low anterior resection, and a colocolic anastomosis was performed. Intraoperatively, a 20x20 cm fleshy mass was seen adhered to the sigmoid colon and upper part of the rectum. Uterus and bilateral ovaries were normal. The resected specimen, along with proximal and distal donuts, was sent for histopathology.

**Figure 2:**
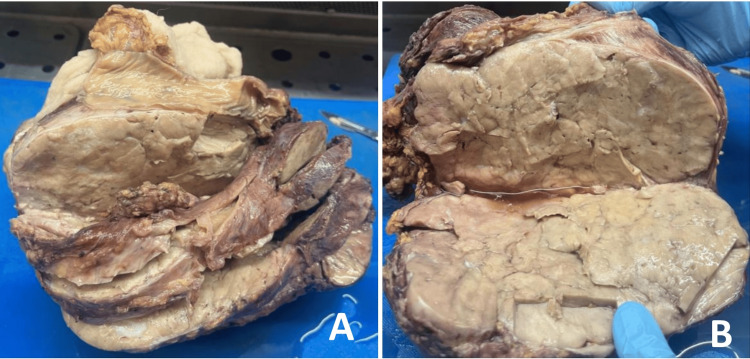
Gross images of the resected specimen. (A) A well-circumscribed grey-white tumour mass attached to the sigmoid colon on the top. (B) A cut section of the tumour shows a grey-white solid surface with a variegated appearance.

Macroscopic findings showed a well-circumscribed grey-white mass measuring 17.5x13x10.5 cm attached to the serosal surface of the sigmoid colon and upper part of the rectum. A cut section showed a solid surface with a variegated appearance (Figure 2).

**Figure 3:**
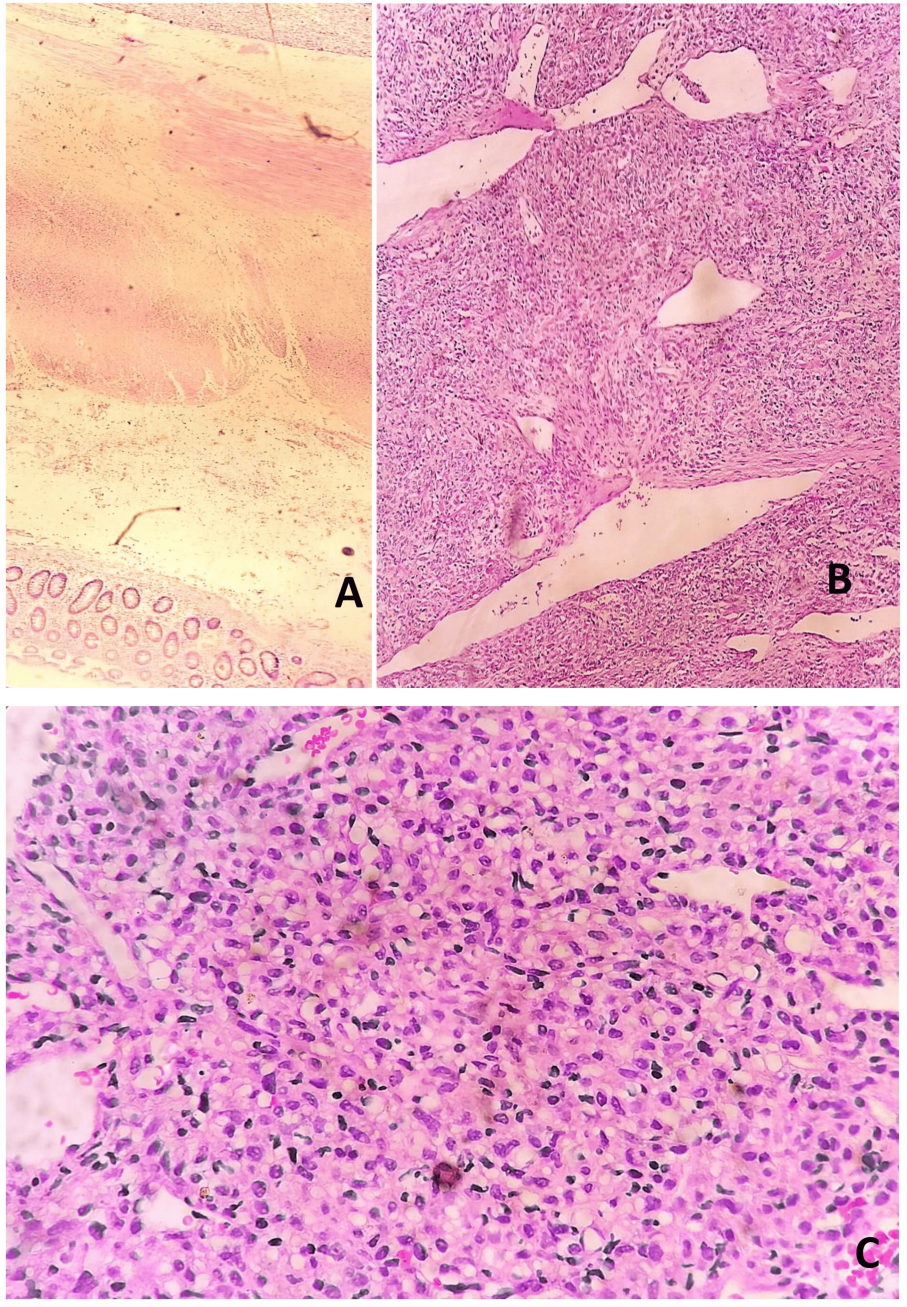
Microscopy (H&E stain). (A) 40x: Colonic epithelium is seen on the lower left corner with the presence of a well-circumscribed tumour attached to the mesentery as seen on the top right corner. (B) 100x: A hypercellular tumour is seen with the tumour cells arranged in haphazard, poorly defined short fascicles along with a vascular network of thin-walled, dilated, and branching blood vessels present between the tumour cells. (C) 400x: Spindle-shaped tumour cells are seen with scant pale eosinophilic cytoplasm, mild to moderate cellular atypia, and vesicular chromatin. Thin-walled, dilated, and branching blood vessels are also seen. H&E: haematoxylin and eosin.

Histopathology showed a hypercellular tumour with the tumour cells arranged in haphazard, poorly defined short fascicles, and storiform pattern. These cells were spindle-shaped with scant pale eosinophilic cytoplasm, mild to moderate cellular atypia, and vesicular chromatin. A vascular network of thin-walled, dilated, and branching blood vessels was seen between the tumour cells. Focal areas of necrosis were also noted. Histopathological and clinical high risk of aggressive behaviour markers, including tumour size, cellular atypia, tumour site, hypercellularity, and necrosis, were noted (Figure 3).

**Figure 4:**
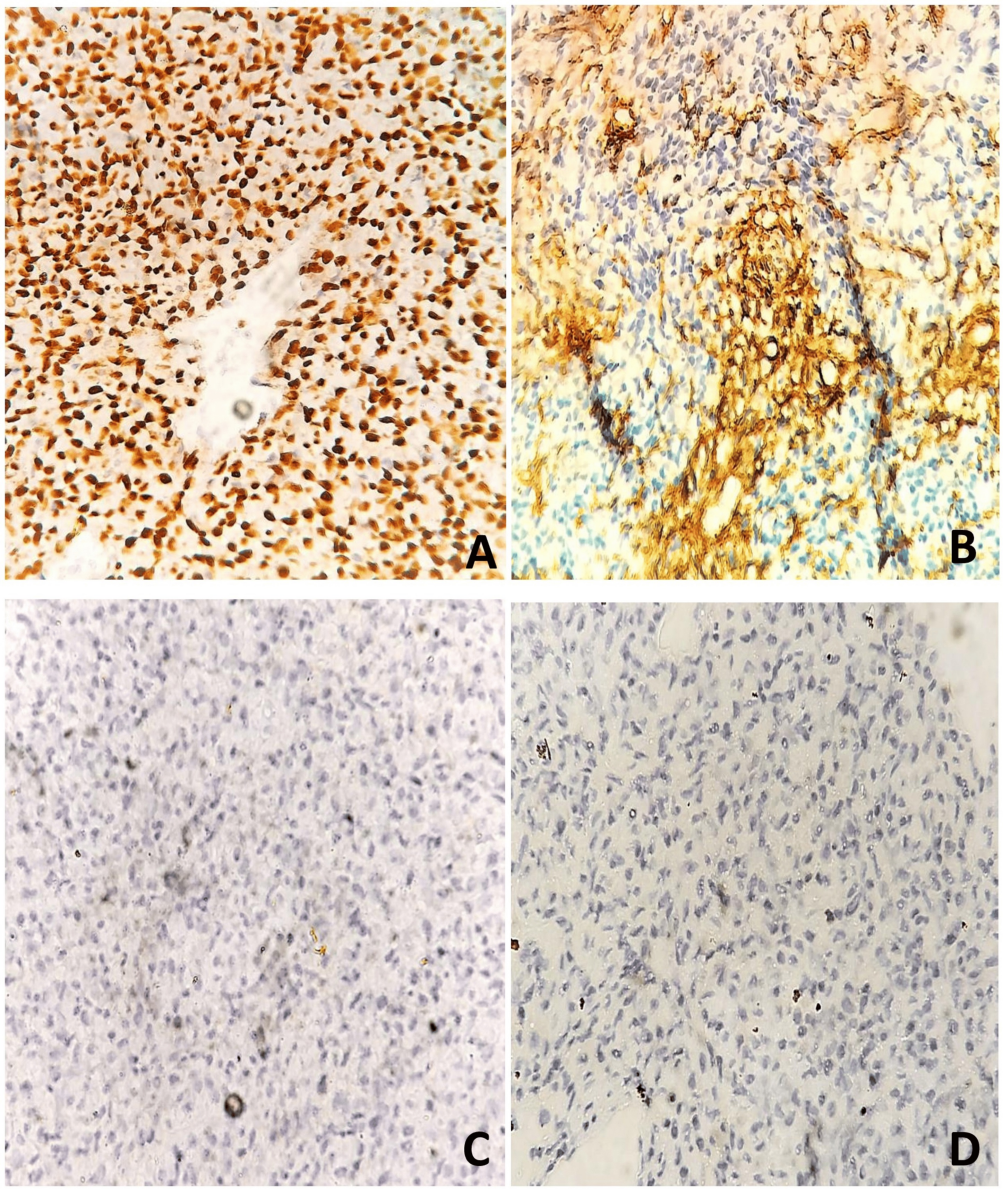
Microscopy: IHC. (A) STAT6 IHC showing nuclear positivity in tumour cells. (B) CD34 IHC showing cytoplasmic positivity in tumour cells. (C) Desmin IHC negativity in tumour cells. (D) CD117 IHC negativity in tumour cells. IHC: immunohistochemistry.

Immunohistochemical staining showed strong and diffuse nuclear positivity for STAT-6 and strong membranous positivity for CD-34. The tumour was negative for DOG1, CD-117, Desmin, Myogenin, MyoD1, SMA, S100, HMB-45, and TLE1. Thus, a diagnosis of SFT was favoured. The patient recovered from the surgery, was asymptomatic at discharge, and was advised to have monthly follow-up or to seek medical attention as soon as possible if any problems arose in the future (Figure 4).

## Discussion

SFT is a rare fibroblastic predominantly affecting the pleura but now found to occur at a wide variety of other sites, including the peritoneum, mediastinum, retroperitoneum, upper respiratory tract, orbit, somatic soft tissue, oral soft tissue, extremities, and almost any other organs [6,7]. Men and women are equally affected by extrapleural SFTs, which are most common in adults, peaking in occurrence between 40 and 70 years [4].

SFT of the mesentery is an extremely rare entity, and a handful of such cases have been reported in medical literature. Fukunaga et al., in their case report, mentioned two such cases and provided information about the 15 cases of SFTs affecting the mesentery that are noted in the literature [8]. The majority of tumours appear as a painless, slowly expanding lump. While head and neck SFTs may manifest as bleeding, vocal abnormalities, or nasal obstruction, abdominal and pelvic tumours may cause distention, constipation, urinary retention, or early satiety [9]. Due to the tumour's synthesis of insulin-like growth factor 2 (IGF2), large SFTs may cause paraneoplastic diseases such as Doege-Potter syndrome, which can manifest as signs of severe hypoglycaemia or acromatoid alterations [10].

Macroscopically, SFTs are well-circumscribed and measure 5-10 cm commonly; however, it can be more than 5 cm in some cases. The cut section is nodular and ranges in colour from reddish-brown to tan, and on rare occasions, it may exhibit cystic degeneration, myxoid alteration, or haemorrhagic areas [4]. Microscopically, SFTs are made up of haphazardly arranged spindled to ovoid cells with a pale, eosinophilic cytoplasm intermingled with branching and hyalinized staghorn-shaped blood vessels (hemangiopericytomatous). Collagenous stroma is present in the background. Low mitotic counts and no significant nuclear pleomorphism or necrosis are the most common characteristics of SFTs [4]. The histological criteria for malignancy include pleomorphism, necrosis, haemorrhagic regions, and a high mitotic count. The tumour might range from a benign tumour to a very aggressive tumour with minimal differentiation (poorly differentiated) [10]. Demicco's classification allows SFTs to be categorized into three levels of malignancy (low, intermediate, and high) based on factors such as age, the number of mitoses, the size of the tumour, and the percentage of necrosis [11]. By immunohistochemistry, SFT typically shows strong and diffuse expression of nuclear STAT6 and CD34. It is negative for S100, actin, desmin, and keratin, which provides valuable diagnostic support.

SFTs are characterized by *NAB2-STAT6* gene fusions at all anatomic locations. However, standard cytogenetics and PCR-based approaches have difficulty detecting the fusion of the *NAB2* and *STAT6* genes because of their close proximity on chromosome 12q. For all fusions, STAT6 immunohistochemistry serves as a precise and sensitive surrogate marker.

The histopathological differential diagnosis includes fibromatosis, GIST, desmoplastic mesothelioma, synovial sarcoma, mesenchymal chondrosarcoma, and dermatofibrosarcoma protuberans in retroperitoneal and pelvic cases. Usually, morphology and careful use of immunohistochemistry can typically provide an accurate diagnosis of SFT [12].

Although most cases of extrathoracic SFTs behave benignly, the prognosis for these tumours is difficult to predict. In fact, approximately 10-36% of these tumours display aggressive characteristics, with local and/or distant recurrences potentially emerging years after the initial diagnosis. Currently, no definitive correlation exists between histological features and tumour behaviour, but malignant histological characteristics, particularly elevated mitotic counts, remain the most reliable indicators of a poor prognosis. The location of the tumour affects how it behaves as well; tumours in the limbs are typically benign, while aggressive tumours are more likely to be found in the mediastinum, pelvis, abdomen, meninges, and retroperitoneum [3]. Additionally, tumours larger than 10 cm and those with positive surgical margins are associated with a more aggressive course. When metastases occurs, the lungs, liver, and bones are the most often affected organs. Thus, thorough clinical follow-up is necessary for all extrapleural SFTs [13].

In our reported case, the diagnosis of SFT presented with a diagnostic dilemma considering the atypical location and absence of any convincing clinicoradiological findings to substantiate our diagnosis. The diagnosis of common tumours affecting the sites was systematically ruled out. Histomorphology served as the initial step to the diagnosis; however, the negativity of various immunohistochemical stains and positivity of STAT6 helped in the exclusion of common neoplasms such as GIST, liposarcoma, dermatofibrosarcoma protuberans, myofibroblastic tumours, synovial sarcomas, and various neural origin neoplasms predominantly affecting the anatomic location. Also, the described case shows the presence of histologically and clinically defined aggressive features, which may bear prognostic and therapeutic implications. No defined standardized treatment regime exists for the treatment of mesenteric SFTs. Nishikawa et al. recommended 50 Gy local irradiation for giant mesorectal SFTs showing partial response [14]. However, the ideal treatment approach still remains an area of debate and active research. The utility of targeted therapeutic options remains to be explored for such neoplasms. In our case, the patient was managed by surgery alone without any radiotherapy, considering no definite guidelines exist for the use of concomitant or follow-up radiotherapy and is planned for aggressive management if recurrence occurs in the future.

The authors highlight this case to shed light on the presence of such rare neoplasm and the utility of immunohistochemistry and histomorphology to aid in the diagnosis and to differentiate it from its mimickers.

## Conclusions

Although SFT is not considered an extremely malignant neoplasm, it can present anywhere and may show aggressive behaviour. It needs to be identified and treated as early as possible for the betterment of the patient. Also, even though the tumour can be entirely resected through surgery, instances with a large tumour and a high mitotic index require close monitoring, as they may develop cancerous potential and thus emphasize the importance of histopathological as well as immunohistochemical findings in such cases. Radiologists and clinicians need to be aware of this entity when evaluating unusually large enhancing masses in the pelvis, and SFTs should be considered in the differential diagnosis of large pelvic tumours.
